# Social Determinants of Health and Depression among African American Adults: A Scoping Review of Current Research

**DOI:** 10.3390/ijerph19031498

**Published:** 2022-01-28

**Authors:** Brooks Yelton, Daniela B. Friedman, Samuel Noblet, Matthew C. Lohman, Michelle A. Arent, Mark M. Macauda, Mayank Sakhuja, Katherine H. Leith

**Affiliations:** 1 Department of Health Promotion, Education, and Behavior, Arnold School of Public Health, University of South Carolina, 915 Greene Street, Columbia, SC 29208, USA; yeltonab@sc.edu (B.Y.); noblet@mailbox.sc.edu (S.N.); mgalardi@mailbox.sc.edu (M.A.A.); macauda@mailbox.sc.edu (M.M.M.); msakhuja@email.sc.edu (M.S.); khleith0@mailbox.sc.edu (K.H.L.); 2 Office for the Study of Aging, Arnold School of Public Health, University of South Carolina, 915 Greene Street, Columbia, SC 29208, USA; lohmanm@mailbox.sc.edu; 3 Prevention Research Center, Department of Health Promotion, Education and Behavior, Arnold School of Public Health, University of South Carolina, 915 Greene Street, Columbia, SC 29208, USA; 4 Department of Epidemiology & Biostatistics, Arnold School of Public Health, University of South Carolina, 915 Greene Street, Columbia, SC 29208, USA; 5 Center for Applied Research and Evaluation, Arnold School of Public Health, University of South Carolina, 915 Greene Street, Columbia, SC 29208, USA

**Keywords:** depression, African American, mental health, social determinants of health, Healthy People 2030, review

## Abstract

Depression in the United States (US) is increasing across all races and ethnicities and is attributed to multiple social determinants of health (SDOH). For members of historically marginalized races and ethnicities, depression is often underreported and undertreated, and can present as more severe. Limited research explores multiple SDOH and depression among African American adults in the US. Guided by Healthy People (HP) 2030, and using cross-disciplinary mental health terminology, we conducted a comprehensive search to capture studies specific to African American adults in the US published after 2016. We applied known scoping review methodology and followed Preferred Reporting Items for Systematic Reviews and Meta-Analyses extension for Scoping Reviews (PRISMA-ScR) guidelines. From 12,315 initial results, 60 studies were included in our final sample. Most studies explored the HP 2030 Social and Community Context domain, with a heavy focus on discrimination and social support; no studies examined Health Care Access and Quality. Researchers typically utilized cross-sectional, secondary datasets; no qualitative studies were included. We recommend research that comprehensively examines mental health risk and protective factors over the life course within, not just between, populations to inform tailored health promotion and public policy interventions for improving SDOH and reducing racial and ethnic health disparities.

## 1. Introduction

Depression, the common reference for a group of depressive disorders [1], is one of the leading global causes of disability [2]. Depression is a major risk factor for suicide [1,3] and poses a serious individual health burden with ripple effects into the community. The prevalence of depression and suicidal ideation among adults in the United States (US) has been steadily increasing [4]. While depression prevalence rates appear relatively similar across races and ethnicities, research suggests greater persistence of mental illness and reduced treatment usage for persons identifying as members of minoritized racial and ethnic groups [5]. Recent data indicate that approximately 17–21% of US adults reported symptoms of depression; approximately 19% of those identifying as non-Hispanic Black reported experiencing symptoms of depression, with approximately 7% reporting moderate to severe symptoms [6]. National reports of depression further increased in the wake of the COVID-19 pandemic. Between 23 April 2020 and 10 January 2022, rates of reported symptoms of depression for non-Hispanic Black participants climbed from 25.6% to a peak of 35.2% in early December 2020 before declining toward initial levels [7].

Healthy People 2030 (HP 2030) aims to ensure “healthy, thriving lives and well-being free of preventable disease, disability, injury, and premature death” and acknowledges the need to achieve health equity through structural and behavioral intervention [8]. HP 2030 defines social determinants of health (SDOH) as, “the conditions in the environments where people are born, live, learn, work, play, worship, and age that affect a wide range of health, functioning, and quality-of-life outcomes and risks”. These are categorized into five domains: Economic Stability, Education Access and Quality, Health Care Access and Quality, Neighborhood and Built Environment, and Social and Community Context. Within these domains are several potential risk and protective factors for mental health outcomes relating to access, quality, and safety of material, social, educational, occupational, civic, and health-related resources [9]. For African Americans, the effects of institutional racism across each of these domains increase the likelihood of psychological distress resulting from inadequate or deleterious conditions in all environments experienced throughout the lifespan. Some basic examples of institutional racism include residential segregation and environmental hazards, discriminatory hiring or policing practices, and reduced access to opportunities and resources in which to build economic capital [10]. Coupled with racial bias and discrimination on a societal scale, the increased stress burden across domains compounds to create unfavorable conditions for mental health.

Comprehensive literature on the influence of SDOH of health on African American mental health, and specifically depression, in the US is somewhat limited. Categorically, several reviews focused on the impact of racism on mental health outcomes among African Americans. Pieterse et al. [11] and Williams and William-Morris [12] focused on mental health more broadly, while additional reviews focused on the association between discrimination and depressive symptoms in Black men [13] and the association between depressive symptoms and perceived discrimination [14]. Further, Watkins et al. [15], Ward and Mengesha [16], and Plowden et al. [17] discussed risk factors for depression in African American men, including several SDOH, and more recent studies have highlighted the negative impact of police encounters [18] and community violence on African American male mental health [19]. A different perspective from Reed et al. [20] explored social work research on SDOH as protective factors regarding African American suicide.

Plowden et al. [17] also postulated depression in African American men may be higher than previously estimated due to hesitancy in treatment seeking and differential symptom presentation. Their research suggests that societal expectations of Black men influence mental health treatment seeking, whereas social and community support may serve as facilitators. Reed et al. [20] echoed this sentiment regarding suicide mitigation, suggesting that social support from family, peers, and the religious community may also mitigate negative effects of perceived racism on African American mental health [11]. This is paramount as greater perceived racism is associated with greater depressive symptoms in African American men [13].

Recognition and assessment of the unique and nuanced social determinants of mental health for African Americans are crucial for effective prevention, diagnosis, and treatment to reduce racial mental health disparities and improve psychological well-being for all. While these studies catalogue important risk and protective factors for African American mental health, to our knowledge, ours is the first scoping review to survey the full field of study on social determinants of depression and depressive symptoms among African Americans in the United States, guided by HP 2030.

## 2. Materials and Methods

Following the methodological framework developed by Arksey and O’Malley [21], and expanded upon by Levac et al. [22], we sought to explore the field of study on the social determinants of mental health specific to the adult African American population in the United States. Guided by the research question, “What social determinants of health are researchers studying in relationship to mental health outcomes among African American adults?”, we developed a search strategy to gather a wide selection of peer-reviewed literature examining our key concepts, inclusive of a variety of study types and disciplines, to assess the volume and scope of this literature.

### 2.1. Search Strategy and Selection Criteria

In consultation with a health sciences librarian, two team members developed a comprehensive search strategy through an iterative process, beginning with a basic draft of search terms for each of our five concepts: (1) social determinants of health, (2) mental health, (3) African Americans, (4) adults, and (5) United States. The librarian and one team member conducted a series of pilot searches in February 2021 to ensure the search results captured the desired concepts. Given the overlap between SDOH with physical and mental health outcomes, some terms were narrowed for relevance to mental health specifically.

The concept of SDOH began with an inventory of key indicators within the five domains of SDOH as outlined in Healthy People 2030 [8]. To ensure concept integrity, these indicators were compared to the SDOH “Z Codes” (Z55-65) in the International Statistical Classification of Diseases and Related Health Problems, 10th Edition (ICD-10) [23], which is used within health systems to document the presence of a variety of psychosocial/economic patient factors. With a comprehensive list of search terms, we matched each to relevant controlled vocabulary and related keyword terms for each database.

The concept of mental health began with a list of broad terms used within the fields of social work, psychology, psychiatry, and nursing (e.g., “mental health”, “behavioral health”, and “well-being”), and some common terms for mental health conditions with high prevalence in the United States, particularly in relationship to SDOH (e.g., “depression” and “anxiety”). These terms were then matched to relevant controlled vocabulary and correlated keyword terms for each database. While some indexed headings were exclusive to clinical nomenclature (e.g., “Mental Disorders” [MeSH]), we did not limit keyword terms to formal diagnoses to account for a range of mental health outcomes, including subclinical or self-described symptomology.

The African American concept largely focused on “race”, with exclusion of studies examining ethnically diverse samples. Despite the limitations of using monolithic constructs of “race”, we chose to focus our sample on studies with samples identifying as having African ancestry or identifying as Black with no other ethnic variation. Since our aims were to survey the field and not necessarily to compare findings, we sought to hone our selection criteria for race and ethnicity to capture as many studies documenting African American experiences with SDOH as possible, while also acknowledging variation in how people self-identify.

One team member conducted a full search in PubMed, PsycINFO, and CINAHL on 26 March 2021. Given the sweeping changes to American life and the disparate socio-cultural-economic and health and mental health impacts of COVID-19 on minoritized populations, the increased media coverage of police brutality toward Black and African American individuals, and the proliferation of prejudiced rhetoric in US politics and on social media, we elected to focus our search on the past five years for timeliness and relevance. Since our search was carried out in early 2021, we also included studies published up until our March 2021 search date, which constituted an additional portion of a sixth year. Studies were eligible if they were (i) peer-reviewed, (ii) empirical articles, (iii) written in English language, (iv) conducted in the United States, (v) published between 2016 and 2021, and (vi) indicated a relationship between one or more SDOH and one or more mental health outcomes. The full search strategy can be found in Appendix B.

### 2.2. Data Extraction and Analysis

**Figure 1:**
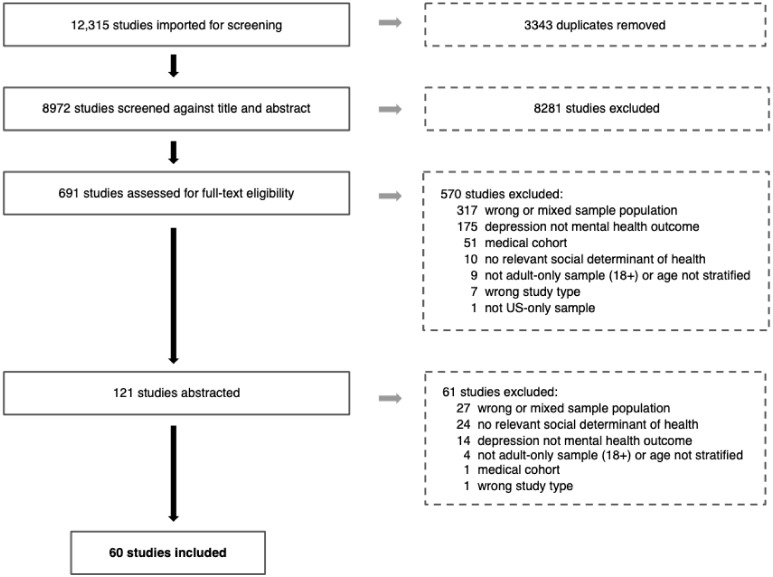
Search and Selection Process.

Once each search was complete, abstracts (*n* = 12,315) were uploaded into EndNote [24] and duplicates (*n* = 3343) were removed. The remaining unique abstracts (*n* = 8880) were then uploaded into the Covidence online content manager [25], and one team member conducted an initial review of title/abstract screening to remove irrelevant articles based on study type, sample population, publication year, and language written, resulting in a sample size of 6124. Our full team was then randomly assigned an equal number of abstracts for review. Assignments were made so that each abstract was reviewed by a blind pair for inclusion using a “yes”, “no”, or “maybe” vote. Two team members developed an inclusion/exclusion rubric to aid efficiency and quality of review, and this rubric was modified through an iterative process with input from the full team for clarity. One team member served as tiebreaker. Given the large number of studies meeting criteria for inclusion in the title/abstract screening phase (*n* = 698), and the increasing prevalence of depression within the US African American and non-Hispanic Black population, the team agreed to narrow selection criteria to exclude (i) studies focused on substance use outcomes, (ii) studies including a multi-racial sample, (iii) studies specific to a medical cohort (e.g., participants with HIV, diabetes, and cancer), and (iv) studies where depression was not a mental health outcome. Two team members were charged with reducing studies based on these criteria through title/abstract screening in Covidence. The resulting sample of studies (*n* = 121) was then randomly divided among all team members for final review and data abstraction utilizing the full article text. Abstraction questions were initially developed by one team member and refined by the full team through an iterative process. Data abstraction from this sample of articles was then conducted using Qualtrics [26]. Chart data were exported into Excel [27] and reviewed by four team members for accuracy. During this process, an additional 61 studies were eliminated, for a final sample size of 60 studies. The full PRISMA chart of results is presented in Figure 1. Descriptive statistics (e.g., frequencies and percentages) were used to examine the abstracted data (Table 1) and summaries of these findings by SDOH domain were compiled by five authors. Three authors summarized relevant study findings associated with depression/depressive symptoms for each study for presentation in the full sample table (Appendix A).

## 3. Results

### 3.1. Sample Characteristics

Search results from 2016 to 2020 ranged from 2120–2565, while search results from 1 January to 26 March 2021 produced 2366 results. The majority of studies examined only depression or depressive symptoms (*n* = 36), while 40% (*n* = 24) of studies also examined another mental health outcome. The ages of participants in the included studies ranged from 18 to 100 years, with an overall mean age of 36.3 years. Of the 60 studies, 25% employed samples consisting entirely of female participants, while 18% employed samples consisting entirely of male participants. Across all 60 studies, the percentage of female participants was 58.5%. The percentage of participants reporting as African American, African American/Black, or Black was 65%, 23.3% and 11.7%, respectively. Of the 60 studies included in this review, 30% employed samples drawn from urban areas, such as large metropolitan centers like Houston, Atlanta, and Chicago. In 12% of studies, samples consisted of college students or of pregnant/new/current mothers, and 5% of studies employed samples consisting of church members. 

### 3.2. Datasets and Depression Measures

The majority of studies in this review utilized secondary data (*n* = 34, 56.7%). Most were cross-sectional studies (*n* = 49, 81.7%), with fewer presenting longitudinal data (*n* = 11, 18.3%). Many studies shared results from unnamed author-developed datasets (*n* = 18, 30.0%) followed by use of the National Survey of American Life (NSAL, 2001–2003; *n* = 11, 18.3%). Three datasets were employed in two studies each (3.3%)—Creating a Higher Understanding of Cancer Research and Community Health (CHURCH, 2012–2013), Nashville Stress and Health Study (NSAHS, 2011–2014), and Religion and Health in African Americans (RHIAA), and twenty-five (25) datasets were used in one study each (1.7%). Most studies focused on depressive symptoms (*n* = 53, 88.3%). One study (1.7%) used the term depression on its own, and one (1.7%) examined both depression and depressive symptoms. The remainder (*n* = 5, 8.3%) examined major depressive disorder or major depressive episode and its connection to SDOH. The majority of studies used established measures, such as the Center for Epidemiological Studies Depression (CES-D) Scale (*n* = 37, 61.7%).

### 3.3. Social Determinants of Health

Studies in our sample examined SDOH from four of the five HP 2030 domains: Economic Stability (*n* = 15, 25.0%), Education Access and Quality (*n* = 10, 16.67%), Neighborhood and Built Environment (*n* = 13, 21.7%), and Social and Community Context (*n* = 47, 78.3%). No studies in the final sample examined a relationship between depression and SDOH listed under the Health Care Access and Quality domain. Across all 60 studies, 108 individual determinants were measured. The majority of studies (*n* = 41, 68.3%) examined SDOH from a singular domain, while 23.3% explored SDOH from two domains, 6.7% examined SDOH from three domains, and one study (1.7%) examined at least one social determinant from all four domains. See Table 1 for SDOH constructs by domain and Appendix A for full study sample data, including outcome summaries.

#### 3.3.1. Economic Stability

The Economic Stability domain comprised 18.5% of the total SDOH studied in our sample. The 20 constructs studied spanned 15 studies (25%) and are categorized as follows: economic hardship/pressure (9 constructs), employment status (4 constructs), subjective social status (2 constructs), income/poverty level (3 constructs), socioeconomic status (SES), and childhood SES. Economic hardship/pressure was the most studied category of the five categories found and was further categorized as follows: financial difficulties (2 constructs), economic strain, economic pressure, financial resources, financial status, financial strain, material hardship, and perceived financial strain. These categories were measured using eight author-developed measures and one scale, the MacArthur Scholar of Subjective Social Status. Of the 15 studies focusing on Economic Stability, 13 studies also examined determinants from one or more additional domains: Education Access and Quality (*n* = 7), Social and Community Context (*n* = 11), Neighborhood and Built Environment (*n* = 3).

#### 3.3.2. Education Access and Quality

Ten studies (16.7%) fell under the Education Access and Quality domain, examining the association between educational attainment and depression or depressive symptoms. The SDOH in this domain comprised approximately 9.3% of the total SDOH studied in our sample and were all associated with educational attainment. These constructs were measured using either categorical scales (*n* = 5) or interval scales (*n* = 5) describing level of education. Of those 10 studies, six also examined Economic Stability determinants, four also examined Social and Community Context determinants, and two also examined Neighborhood and Built Environment determinants.

#### 3.3.3. Neighborhood and Built Environment

The Neighborhood and Built Environment domain comprised 16.7% of the total SDOH studied, with 18 total constructs spanning 13 studies. These SDOH were further categorized as: neighborhood disorder (7 constructs), neighborhood cohesion/participation (4 constructs), intimate partner violence (3 constructs), neighborhood vigilance, neighborhood income, and community racism. The neighborhood disorder category was composed of the following author-defined determinants: perceived neighborhood conditions (2 constructs), neighborhood problems (2 constructs), neighborhood disorder, social disorder, and residential environment. These determinants were measured by several composite scales including the Neighborhood Assessment Scale, the Perceived Neighborhood Disorder Scale, and five author-developed scales assessing a variety of neighborhood factors: transportation, quality of schools, police protection and tension, safety (crime/violence), drug use and dealing, walking environment, park access, healthy food availability, social disorder, vacant/deserted buildings, litter, vandalism, and noise, and neighborhood trust/willingness to help. Six studies examined this domain alone, while seven studies also examined SDOH within the Economic Stability (*n* = 4), Education Access and Quality (*n* = 1), and Social and Community Context (*n* = 6) domains.

#### 3.3.4. Social and Community Context

The Social and Community Context domain comprised 56.5% of the total SDOH studied in our sample. These 61 constructs spanned 47 studies and are categorized as follows: discrimination (30 constructs), social support (24 constructs), incarceration/criminal justice contact (4 constructs), negative police encounters (2 constructs), and living arrangement.

Discrimination was further categorized as follows: everyday discrimination (*n* = 17, 56.67%), composite measure (*n* = 5, 16.7%), early life discrimination (*n* = 3, 10.0%), past/lifetime discrimination (*n* = 3, 10.0%), adult discrimination (*n* = 1, 3.3%), and sexual-racial discrimination (*n* = 1, 3.3%). These constructs were studied via the following measures: Everyday Discrimination Scale (*n* = 10) and Lifetime Discrimination Scales (*n* = 1); Perceived Experiences of Discrimination scale (*n* = 2); Racism and Life Experiences (RLE) scale (*n* = 2), Daily Life Experience subscale (*n* = 4), Experiences of Discrimination subscale (*n* = 2), and Major Discrimination subscale (*n* = 1); the Schedule of Racist Events (*n* = 1) and General Ethnic Discrimination subscale (*n* = 1); 10-item subset of the National Survey of American Life survey (*n* = 1); Day-to-Day Unfair Treatment Scale (*n* = 1); Perceived Ethnic Discrimination Questionnaire–Community Version (*n* = 1); author-developed questions (*n* = 3); other (*n* = 1). Approximately 81% (*n* = 22) of studies reported a direct positive association between discrimination and depression/depressive symptoms, and 9% (*n* = 5) of studies reported mixed results.

Social support was the second most frequently studied category (24 constructs), and was author defined as follows: social support (11 constructs), perceived social support (3 constructs), support from family (2 constructs), religious social support (2 constructs), conflict with partner, family involvement, frequency of social contact, interpersonal relationship stress, relationship quality, and social resources. Social support was measured via 12 author-developed instruments, and Medical Outcomes Study Social Support survey (*n* = 3), Arizona Social Support Interview Schedule, Interpersonal Support Evaluation List, modified Perceived Social Support Scale, modified Social Network List, Multidimensional Measurement of Religiousness/Spirituality for use in Health Research, National Survey of American Life subscale, Older Americans Resources and Services Assessment, Provisions of Social Relations scale, and Social Support Behaviors Scale.

Thirty-three (*n* = 33) studies only explored this domain, while 10 studies also examined Economic Stability determinants, six studies also examined Neighborhood and Built Environment determinants, and five studies also examined Education Access and Quality determinants.

## 4. Discussion

This is the first scoping review to examine in depth how SDOH, as categorized by HP 2030, are being studied in relation to depressive symptoms and depression outcomes among African American adults in the US. The larger proportion of young adults in our full sample is consistent with increasing and higher rates of depression among adults 18–29 years old. Depression rates are lowest among those 30–44 years old, while somewhat higher for those 45 years and older [6,28]. In addition, the slight majority female composition of our full sample is consistent with higher rates of depression for women in the US at all levels of symptom severity [7].

### 4.1. Social Determinants of Health

Most studies examined the relationship between depression and SDOH under the HP 2030 Social and Community Context domain. No studies in the final sample examined a relationship between depression and SDOH within the Health Care Access and Quality domain.

#### 4.1.1. Economic Stability

Only 4 of the 14 studies involving SDOH under this domain focused solely on Economic Stability, suggesting that researchers often consider socioeconomic factors in conjunction with other SDOH. This domain was stratified by the subcategories economic hardship/pressure, employment status, subjective social status, income/poverty level, and childhood SES. Economic hardship/pressure was the most studied subcategory and was mostly measured by individuals’ self-reported ability to meet their basic needs on a regular basis. This dimension of Economic Stability reflects perceived current financial strain, which objective ordinal and ratio measures do not convey, and may better predict increased risk of depression [29]. For example, while income and household poverty level are also associated with depression [29,30], causation is less clear, and these indicators may be offset by federal assistance programs that improve ability to meet basic needs. Additionally, persons with a higher income or improved position in relation to poverty level may experience greater perceived economic hardship/pressure depending on their costs of living, debt, and lifestyle preferences. While nuances of causation vary or are unclear, research demonstrates an association between employment status and depression [31,32] and between childhood SES and adult depression [33,34]. Research on subjective social status is mixed, however, and may not as strongly predict depression for African Americans [35]. Discrimination and other systemic factors may reduce incremental benefits of gains in socioeconomic mobility [35,36,37]. All factors considered, Economic Stability is one of the most influential social determinants of health and mental health across races and ethnicities [38,39] and is of importance regarding the African American and Black experience due to the historic wealth and wage gap and high levels of poverty and unemployment resulting from structural racism [39,40].

#### 4.1.2. Education Access and Quality

Education Access and Quality was the least studied domain, with 15% of our total sample examining the association between educational attainment and depression or depressive symptoms; only one study examined educational attainment as the sole determinant, exploring the differential impact of educational attainment on depressive symptoms for men and women [41]. Educational attainment is often considered an indicator of SES, but research demonstrates an individual association between education level and depression [42,43,44]. Childhood SES also has a tremendous impact on educational attainment through a variety of pathways [45,46,47], and higher educational attainment is impacted by a variety of economic and social and community factors. It is not surprising that educational attainment was the only social determinant in our final sample related to the broader category of Education Access and Quality, as both education access (e.g., school choice, availability of early education, language assistance, admissions and affordability of higher education) and education quality (e.g., school resources, teacher-to-student ratios, special education services, teacher education level, college preparatory classes, and guidance counselors) impact educational attainment. There is a large body of research demonstrating the impact of systemic racism and residential segregation on quality of education, with obvious historical roots to inequitable access to education dating well beyond *Plessy v. Ferguson* and Jim Crow segregation [48,49,50]. More research is warranted on specific aspects of Education Access and Quality on African American adult mental health to determine if there are micro-effects within this domain, or if educational attainment is the sole variable for depression.

#### 4.1.3. Neighborhood and Built Environment

The Neighborhood and Built Environment domain was the most diverse regarding author-defined constructs. This domain comprised 17% of total constructs studied, distributed across 13 studies, with more than half of studies focused solely on the Neighborhood and Built Environment domain. Neighborhood disorder was the most studied subcategory, followed by social cohesion. Many of the measurement constructs for neighborhood disorder, however, overlapped with those of SDOH in other subcategories, as disorder was treated as a composite construct. Research demonstrates an association between depression and both perceived neighborhood disorder and social cohesion across races and ethnicities [51,52]. Evidence is mixed for associations between the less frequently studied subcategories of perceived neighborhood safety (e.g., vigilance and violence) and neighborhood income and depression across races and ethnicities [52,53,54,55]. It is increasingly important to recognize the potential mental health impacts of neighborhood disorder for adults identifying as African American or Black, as redlining and other historical contributors to segregation have had a disparate impact on neighborhood choice and mobility [56]. Residential segregation has been associated with cumulative neighborhood disadvantages, which increase risks for negative health and mental health outcomes [57,58].

There were two SDOH studied in our sample that straddle both the Neighborhood and Built Environment and Social and Community Context domains: community racism and intimate partner violence (IPV). Community racism, measured in the original study in aggregate at the neighborhood level, could fall within the discrimination category, listed in HP 2030 under Social and Community Context. It is interesting, however, to consider neighborhood-specific racism a unique variable to be studied both individually and in the context of other measures of racial discrimination (everyday experiences, historical experiences, etc.). Additionally, IPV is only mentioned within the “Crime and Violence” literature summary for HP 2030, which suggests that this is a key issue within the Neighborhood and Built Environment domain. IPV occurs within a dyad relationship with direct, indirect, and intergenerational spillover effects on families, households, friends, neighbors, and community members; thus, this could be considered an important social and community construct, particularly in the context of social contagion [59]. Research suggests that neighborhood environment may influence the potential for IPV, however, through a variety of macro-, meso-, and exo-level pathways [60,61]. The term “community” is very broad, applied to geographic, political, cultural, and social groups, whereas “neighborhood” is specific to a residential area. The authors of this study have listed IPV under Neighborhood and Built Environment to remain consistent with the guiding principles of HP 2030, but argue this type of violence could also be situated within the Social and Community Context domain.

#### 4.1.4. Social and Community Context

The vast majority of studies (77%) explored SDOH housed under this domain, and approximately 72% of these studies explored this domain alone. Discrimination (49.2%) and social support (39.3%) were the most studied subcategories, while five studies explored SDOH related to law enforcement, including incarceration, negative police encounters, and a composite criminal justice contact construct. One study examined the association between living arrangement and depressive symptoms within a population of economically disadvantaged older adults, using it as a proxy for potential social isolation and lack of social support or sense of belonging; however, we included this as a separate social determinant as those elements were not explicitly investigated [62].

It is not surprising that discrimination was the most studied SDOH in our sample, as discrimination has been tied to many negative health and mental health outcomes and is well represented in the literature [58]. More than half of our sample studying discrimination utilized measures of everyday discrimination, followed by those using a composite measure of various forms of discrimination, and measures assessing early life discrimination, past or lifetime discrimination, adult discrimination, and sexual-racial discrimination. Williams et al. [58] note many limitations in measuring discrimination (e.g., capturing chronicity, recurrence, severity, and duration, and traumatic vs. non-traumatic experiences) and recommend expanding the study of discrimination to better understand intersectionality and the cumulative impacts of layered experiences of discrimination across domains and contexts. Given the pervasive nature of discrimination, we argue discrimination should be considered a systemic factor that impacts all domains on institutional, interpersonal, and individual levels, and listing discrimination as a “key issue” within the HP 2030 Social and Community Context domain [63] is highly reductive. To approach equity in public health, research and policy must fully recognize the impacts of discrimination on individuals and communities at each level of the ecological system [58,64,65]. Thus, it should also follow that HP 2030 include discriminatory and aggressive policing as a SDOH in addition to incarceration [66]. Evidenced by the Black Lives Matter movement, there has been public outcry over the differential treatment, mortality, and portrayal in media coverage of African American and Black victims of violence [67]. As previously mentioned, research demonstrates an association between police encounters and officer-involved shootings and negative mental health outcomes for those identifying as African American and Black [18,66,68,69]. Research evidence of these associations is backed by myriad anecdotal accounts in social and news media. Both studies in our sample examining contact with law enforcement found a positive association with depressive symptoms [70,71].

Social support was also frequently studied and was mostly measured via composite constructs of general social support (multiple sources), with other studies examining social support specific to family, frequency of social contact, conflict with a partner, and religious social support. Research demonstrates a strong association between perceived social support and mental health, particularly depression [72,73]. Social support confers tangible, emotional, and informational benefits that can influence psychological well-being, self-esteem, treatment seeking and adherence, and recovery [72,73]. While there may be wide individual variation in preferred sources of social support and relative influence of different sources of social support, research suggests that both family (and fictive kin) and congregational support are important sources within the African American social network [74,75]. Further, spirituality and religious involvement influence health and mental health [76,77,78] and should be considered a SDOH. With 78% of those identifying as African American or Black reporting religious affiliation, and 97% reporting belief in God or a higher power [79], this is a salient area of research regarding African American and Black mental health and well-being. Research has been conducted on spirituality/religious involvement and depression within this population [77,80,81]; however, we did not include these constructs in our study as they are not listed as HP 2030 SDOH.

#### 4.1.5. Health Care Access and Quality

A lack of studies focused on Health Care Access and Quality suggests that insurance coverage for and access to mental health care services and resources may not be current priorities of research on social determinants of depression among African Americans. Indeed, HP 2030 guidance for this domain does not include any objectives specifically related to access to mental health services, other than those for drug and alcohol use disorders. Yet, previous studies have found that despite policies implemented in 2008 and 2010 to promote parity in benefits for mental and physical health services [82,83], insurance coverage for mental health disorders still lags far behind those for physical conditions [84]. This disparity is even more pronounced among individuals identifying as African American or Black who are less likely than White individuals to receive or initiate mental health care [85,86,87]. Racial differences in mental health care utilization may reflect poorer insurance coverage and access, cultural stigmatization of mental health disorders, mental health literacy, economic concerns, lack of racial and ethnic representation among providers, or several other factors [77,85,88]. Regardless of the causes, these persistent disparities warrant increased research attention in the Health Care Access and Quality domain to mental health services and outcomes, with specific attention to depression. In addition, racial and ethnic biases in health care serve to further deter help-seeking and perpetuate stigma and can lead to misdiagnosis and inappropriate or inadequate treatment [85,89,90]. This corrosion of mental health care quality presents a dangerous inequity for minoritized individuals and is of critical importance regarding the alarming rise in African American and Black youth suicide rates [91,92].

### 4.2. Limitations

This study has some limitations. Terminology related to both SDOH and mental health is highly variable, potentially leading to missed studies based on our search strategy. In addition, HP 2030 is not fully developed, so important studies with tangential but relevant concepts (e.g., “internalized racism”, “racial and ethnic identity and centrality”, “religious involvement”) were excluded based on lack of inclusion in the currently available HP 2030 literature. Studies may have been missed during the initial title/abstract screening phase due to vague abstracts lacking relevant keywords. Due to our focus on adults identifying as African American and non-Hispanic Black within the United States, we cannot comment on the scope of literature related to other racial and ethnic groups within or outside of the United States. The lack of results within the Health Care Access and Quality domain may be due to exclusion of studies exploring efficacy of or adherence to mental health treatment services, as we only included studies examining a direct relationship between a social determinant of health and depression/depressive symptoms. In addition, while access to service and quality of services are both SDOH, service use itself is a behavior and not SDOH.

Further, the authors of this study acknowledge our privilege and positionality as a research team and recognize the potential for bias in designing our study and selecting and interpreting results. Four authors on our research team identify as non-Hispanic White females, three authors identify as non-Hispanic White males, and one member identifies as an Asian male. We also acknowledge several factors regarding race and ethnicity that may have led to inaccuracies in our inclusion/exclusion process: (1) race is a social construct that is potentially variable within different social, cultural, and institutional contexts; (2) there is wide variation in how researchers collect racial and ethnic data; thus, race and ethnicity may be misattributed or disregarded in certain samples; (3) despite the importance of research regarding multiracial participants [93], there is wide variation in how researchers collect and report this information. We also excluded studies comparing races and ethnicities. While this type of research can highlight patterns in racial and ethnic health disparities, we viewed this scoping review as an opportunity to survey literature specific to African American and non-Hispanic Black adults. We also recognize that the social construction of race in the US inherently privileges and disadvantages individuals and groups based on the color of their skin. Thus, we argue studies should make an effort to include ethnically diverse samples and to explore both within-group and aggregate associations for a more comprehensive understanding of SDOH, systemic racism, and risk and protective factors for individuals and communities. Since embarking on this scoping review, guidance for reporting of race and ethnicity in journal articles has been published in the *Journal of the American Medical Association* and it is strongly recommended that authors be inclusive in reporting of demographics and provide a comprehensive list of categories, and include categories for participants who may identify with more than one race and ethnicity [93].

### 4.3. Implications and Future Directions

Although this review had limitations, it provides a comprehensive examination of the volume and scope of work examining SDOH and depression outcomes among African American adults. The volume of studies in our sample focused on discrimination (namely, racism) and social support demonstrate acknowledgement by researchers of the importance of these issues in relation to depression amongst African American adults. Mental health care providers typically include social support in assessment and treatment planning, and careful, non-assumptive consideration of individually preferred sources of support, inclusive of faith-based communities and fictive kin, is warranted. At the structural level, it is necessary for policymakers, health care administrators, workforce educators, and clinical providers to recognize systemic racism and denounce White supremacy of any form. All health and mental health practitioners should understand the potential impacts of racism and other forms of discrimination on patient mental health and convey receptivity to relevant patient-led discussions in assessment and intervention. In addition, it is critical mental health care providers of all disciplines examine personal biases, frequently evaluate their practice, and engage in cultural agility trainings to reduce the potential for discrimination in the mental health care encounter. Improving perceptions of mental health care across races and ethnicities and reducing stigma across disciplines is necessary to encourage prevention and intervention for historically under-treated populations [85,88].

Findings also demonstrated researcher attention to economic and neighborhood environment factors, while less attention in our sample to the Education Access and Quality domain could indicate an area of further study, particularly regarding literacy. In addition, both health and mental health literacy (listed by HP 2030 under Health Care Access and Quality) are of increasing importance and present an opportunity for intervention by all types of mental health care providers and at multiple levels of the health care system. For example, partnerships between academic researchers, public health practitioners, clinical providers and administrators, and community literacy centers can serve to improve the patient-provider encounter and facilitate patient referral for literacy supports [94]. Overall, accounting for the influences of SDOH on depression and other mental health sequelae is crucial for improved mental health outcomes, and should become standard practice in all health and mental health encounters. Increased adoption of ICD-10 “Z codes” within the health care system can result in more effective mental health treatment and better inform public health intervention and health care policy [23].

Moving forward, it will be important to expand this type of review to additional mental health outcomes and encourage scholars to assess intersectionality for a more nuanced understanding of SDOH, systemic racism, and risk and protective factors for diverse individuals and communities. While the studies included in this review used rigorous methodologies and representative data sources, they also examined the issues of depression/depressive symptoms and SDOH in very similar ways. We recognize the importance of national datasets in the study of mental health [95]; however, we suggest that there are additional methods that could be used to further understand this issue from other perspectives. For example, over half of the studies reviewed relied on pre-existing data sources, with several using the same national dataset, the National Survey of American Life, which ceased data collection in 2003, thirteen years before the publication date of our earliest studies. Studies also primarily utilized cross-sectional methodology, which does not account for the impact of SDOH on depression across the life course.

Studies that examine in depth the experiences of African Americans within their communities and/or at the neighborhood level would provide rich information about context and the linkage of the SDOH and depression outcomes examined in this study. Qualitative research may explore this; however, if there was not an explicit association between a SDOH under study and depression/depressive symptoms, then this research would not have met criteria for inclusion in our review. A community-level methodology that provides this perspective is photovoice—a methodology that uses photography as a tool to help individuals, especially individuals in populations that might otherwise not have a voice in policy development or decision-making, to document their lived experiences and ensure the research being done is meaningful for their communities. Through a participatory framework, the process promotes dialogue and issue selection with the goal of engaging social change and action. Through discussion about the stories behind the photographs, photovoice has the potential to promote critical dialogue about important community issues such as SDOH related to mental health outcomes [96] and may provide more meaningful information on how SDOH interact in reality. Studies utilizing community-engaged and community based participatory research practices may also foster openness among African American community members to engage in research that will increase the visibility of depression and other mental health issues with the potential to reduce stigma, increase mental health literacy, and promote help-seeking. A more community-driven approach to mental health research in African American communities can also inform how community leaders and members view the HP 2030 categories and classification of SDOH for purposes of practice and policy interventions. In addition, analysis of the HPS found individuals identifying as non-Hispanic Black reported acute socioeconomic stressors due to effects of the pandemic [7,97]. While the COVID-19 pandemic created wide-ranging global stressors across racial, ethnic, and socioeconomic groups, the acute, amplified effects of these stressors on a variety of mental health outcomes for historically marginalized groups should be examined [98].

## 5. Conclusions

This scoping review highlighted the reliance on secondary and cross-sectional research, and a heavy research focus on the relationship between depression and depressive symptoms and SDOH within the HP 2030 Social and Community Context domain, with specific attention to discrimination and social support. As racism and the residual effects of COVID-19 continue to dominate the national conversation on health equity, we recommend research that comprehensively examines mental health risk and protective factors within, and not just between, populations to allow tailored health promotion and public policy interventions to improve SDOH and reduce racial and ethnic health disparities in the US.

## Figures and Tables

**Table 1:** Social Determinants of Health Studied.

Domain	Number of Constructs	Percent of Total Constructs	Number of Studies	Percent of Total Studies
Economic Stability	20	18.5%	15	25.0%
economic hardship/pressure	9			
employment status	4			
subjective social status	2			
income/poverty level	3			
socioeconomic status (SES)	1			
childhood SES	1			
Education Access and Quality	10	9.3%	10	16.7%
educational attainment	10			
Neighborhood and Built Environment	18	16.7%	13	21.7%
neighborhood disorder	7			
neighborhood cohesion/participation	4			
intimate partner violence	3			
neighborhood violence	1			
neighborhood vigilance	1			
neighborhood income	1			
community racism	1			
Social and Community Context	61	56.5%	47	78.3%
discrimination	30			
social support	24			
incarceration/criminal justice contact	4			
negative police encounters	2			
living arrangement	1			
Number of Domains Studied			Number of Studies	Percent of Total Studies
1			41	68.3%
2			14	23.3%
3			4	6.7%
4			1	1.7%

## Data Availability

Not applicable.

## Appendix A

**Table A1:** Full Sample Attributes and Summaries of Relevant Findings.

First Author	Year	Type of Study	Demographics (Race/Ethnicity, Female n/%, Age Range, Mean Age)	Other Demographic Characteristics	Social Determinants of Health	Summaries
Assari [41]	2018	Cross-sectional	AA; 56%; 18+; 42.01		educational attainment	Greater educational attainment was associated with lower depressive symptoms for African American adults, but the effect differed between men and women. Educational attainment reduced depressive symptoms to a greater degree for women than men.
Evans [62]	2020	Cross-sectional	AA; 64.1%; 55+; 71.7	the sample came from Service Planning Area 6, one of the most economically disadvantaged urban areas in Los Angeles County as well as the highest percentage of AAs (27.4%) of the SPAs in Los Angeles.	financial difficulties	Financial difficulty was positively correlated with depressive symptoms.
Bowleg [70]	2020	Cross-sectional	AA, Black; 0%; --; 30.25	Washington DC	incarceration history, police encounters, police avoidance, employment status	Incarceration history had a significant indirect effect on depressive symptoms via negative police encounters and police avoidance. Those with a history of incarceration who were unemployed reported higher depressive symptoms due to higher police avoidance; however, unemployment did not mediate the indirect effect of police encounters on depressive symptoms.
Archibald [71]	2018	Cross-sectional	Black; 56%; 18+; 43.1		criminal justice contact (unfairly stopped, searched, questioned, physically threatened or abused by the police; history of arrest or incarceration; currently on parole)	Criminal justice contact was significantly positively associated with depressive symptomatology and the association of criminal justice contact with depressive symptomatology was attenuated after adjustment for the effects of stress.
Alexander [99]	2019	Cross-sectional	AA, Black; 100%; 18–24; 21.3	young, female, Baltimore MD	intimate partner violence	68.5% of women with IPV experiences reported symptoms indicative of depression relative to 28.1% of women without IPV. Adjusting for covariates, IPV remained significantly and independently associated with greater depression.
Amutah- Onukagha [100]	2017	Cross-sectional	Black; 100%; 20+; 42		poverty income ratio, education attainment	When controlling for other variables, Black women below 299% FPL were close to three times more likely to report major depressive disorder (MDD) symptoms compared to those above 300% FPL (OR = 2.82, 95% CI = 1.02, 7.96). Lower average MDD symptoms were found among those with higher levels of education.
Assari [101]	2020	Cross-sectional	AA, Black; 65.30%; 65+; 74	South Los Angeles; all had hypertension	economic strain, education attainment	Economic strain, but not educational attainment, was associated with depressive symptoms.
Assari [102]	2018	Cross-sectional	AA; 0%; 18+; 41.76	“high” socioeconomic status-AA men	income, employment status, education attainment, perceived discrimination	Perceived discrimination was a risk factor for a major depressive episode (MDE), regardless of covariates; however, high income was associated with an increased risk of a 12-month major depressive episode for African American men independent of perceived discrimination and other SES indicators.
Assari [103]	2018	Cross-sectional	AA; 0%; 18+; 41.76		incarceration, everyday discrimination	History of incarceration among African American men was associated with greater depressive symptoms. There was also a positive and significant association between discrimination and depressive symptoms.
Baldwin- Clark [104]	2016	Cross-sectional	AA, Black; 100%; 50+; --	community dwelling, female, at least 50 years old	receiving support from family	Respondents who received help from relatives were 23.4% (odds ratio = 0.766) less likely to have felt depressed in the past year than people who had received no help from relatives. No other measures of social support had a significant association.
Benca- Bachman [105]	2020	Cross-sectional	AA; 59%; 22–92; 49.86	aging AA twins	social support	Higher satisfaction with emotional social support (i.e., perceived quality) predicted fewer perceived depressive symptoms, but more emotional social support predicted more depression symptoms.
Bottomley [106]	2017	Longitudinal	AA; 89.4%; 19–71; 49.65	recruited from grassroots victim services organization with faith-based orientation in a large city in the mid-South	social support	Compared with five other social support domains (intimate interaction, material aid, advice, positive feedback, and social participation), higher satisfaction with the domain of physical assistance predicted lower depression at a later time.
Brooks [107]	2020	Cross-sectional	AA, Black; 60.4%; 18–38; 21.41	Southwestern US university in introductory psychology class	perceived racial discrimination	Perceived racial discrimination was significantly associated with greater depressive symptoms.
Bukowski [108]	2019	Cross-sectional	AA, Black; 100%; --; 30.9	Recruited from Black Pride events in 6 cities: Atlanta GA, Detroit MI, Houston TX, Memphis TN, Philadelphia PA, Washington DC	intimate partner violence, perceived social support	There was a statistically significant direct effect of intimate partner violence (IPV) on greater depression, with a 36% increase in likelihood for higher depression symptoms among those reporting past year IPV. Each unit of perceived social support, however, reduced likelihood of depression symptomology by 20%.
Caldwell [109]	2018	Cross-sectional	Black; 50%; 18–54; --	expectant mother–father dyads; recruited through prenatal clinics at two local Midwestern sites	conflict with partner, social support	Higher levels of conflict with partner and lower levels of social support were significantly associated with higher depressive symptoms for both mothers and fathers.
Chae [110]	2017	Cross-sectional	AA; 0%; --; 43.8	Bay Area Heart Health Study—San Francisco Bay Area—2010	racial discrimination	There was no significant overall association between discrimination and depression. However, among AA men with an implicit pro-Black bias, there was a positive association between reports of racial discrimination and the probability of having higher depressive symptoms. Among AA men with implicit anti-Black bias, there was a negative relationship between reports of racial discrimination and the probability of having elevated depressive symptoms.
Chang [111]	2019	Cross-sectional	AA; 79.92%; 18–48; 21.58	Eastern public university	racial discrimination, social support	Racial discrimination was significantly positively correlated with depressive symptoms. Family social support and friend social support were significantly negatively correlated with depressive symptoms.
Chatters [112]	2018	Cross-sectional	AA; 58.31%; 18–93; 43.1	attendants of religious services living in the Southern US	social support from church members, social support from family members	Frequency of contact with church and family and emotional support from family were inversely associated with depressive symptoms. Negative church and family interactions were significantly positively associated with depressive symptoms. Emotional support from church was unrelated to depressive symptoms.
Christie- Mizell [113]	2019	Longitudinal	AA; 51.69%; 40–50; --		employment status	AA men who were employed only or married/cohabitating employed parents had significantly lower levels of depressive symptoms compared with AA women. Role combinations of married/cohabitating and employed and married/cohabitating employed parent led to lower probability of clinical depression for AA men compared with AA women.
Clark [114]	2018	Cross-sectional	AA; 53%; 21–92; 56	not having a cancer diagnosis, as those persons were already being recruited for another part of this initiative	social capital	There was a significant positive slope in the relationship between social capital and depressive symptoms for participants regardless of level of neuroticism, indicating that as social capital increased, levels of depressive symptomatology increased.
English [115]	2020	Cross-sectional	AA, Black; 0%; 18–62; 30	Black gay, bisexual, and other sexual minority men and transgender women, living in Atlanta, GA and Jackson, MS	racial discrimination, sexual racial discrimination	Any discrimination and sexual racial discrimination were positively associated with depressive symptoms. There was a significant indirect effect of racial discrimination on depressive symptoms through Black sexual exclusivity and sexual racial discrimination.
Evans [116]	2019	Cross-sectional	AA; 64.1%; 55+; 71.7	residents of Service Planning Area in Los Angeles, identified as one of the most economically disadvantaged urban areas in Los Angeles County, with the lowest median income ($ 36,400), the highest unemployment rate (13.6%), and the highest percentage of household incomes less than 100% of the FPL (33.6%)	financial difficulties, educational attainment, living arrangement	Financial difficulties were associated with depressive symptoms. Educational attainment and living arrangement were not associated with depressive symptoms.
Gayman [117]	2018	Cross-sectional	AA; 0%; 18–86; 58.1		socioeconomic status, neighborhood income, daily discrimination, perceived social support	Increase in neighborhood level income was associated with decreased depressive symptoms (SE = 0.00, *p* ≤ 0.01). Increased depressive symptoms were associated with higher levels of chronic stressors and daily discrimination, but were relatively lower among African American reporting more family support. Family support had the strongest correlation with depressive symptoms (β = −0.48, SE = 0.89, *p* ≤ 0.001) among assessed factors.
Goodwill [118]	2021	Cross-sectional	AA; 0%; 18–93; 43.5		everyday discrimination (any, race-based, other)	Only race-based everyday discrimination demonstrated a significant association with increased depressive symptoms.
Hart [119]	2021	Longitudinal	Black; 50.4%; 21–83; --	participants were families participating in a family centered intervention	racial discrimination	Racial discrimination was positively associated with depressive symptoms for all family members (fathers, mothers, and youth).
Hawkins [120]	2020	Cross-sectional	AA, Black; 100%; 18–42; 26.9	pregnant women	family involvement	Higher family involvement during pregnancy was associated with lower depressive symptoms among pregnant women.
Heldreth [121]	2016	Cross-sectional	AA Black; 100%; 18–40; 24.08	mothers 1 month postpartum	childhood direct discrimination, childhood vicarious discrimination	Direct and vicarious childhood racism experiences were each independently associated with greater postpartum depressive symptoms.
Hoggard [122]	2019	Cross-sectional	AA; 0%; 18+; 32.35		racial discrimination	Racial discrimination was significantly associated with depressive symptoms.
Hoggard [123]	2019	Cross-sectional	AA; 59.3%; 18–27; 20.25	attending a large predominantly White public institution in the southeastern US	racial discrimination	Increased frequency of racial discrimination experiences was significantly associated with greater depressive symptomatology, but only for African Americans with mean or high levels of emotional eating.
Holmes [124]	2020	Cross-sectional	AA, Black; 100%; --; 33.57	women (mothers) receiving TANF	economic pressure, social support	Among Black female primary caregivers who receive Temporary Assistance for Needy Families, economic pressure was associated with maternal depression. Social support was associated with lower levels of maternal depression but did not attenuate the relationship between economic pressure and depression.
Holt [125]	2018	Longitudinal	AA; 59.55%; --; 58.72		religious social support	Positive religious support was associated with lower depressive symptoms, while negative interaction predicted increases in depressive symptoms.
Hudson [126]	2016	Cross-sectional	AA; 56.1%; 18–90; 42.5		racial discrimination	Increased levels of racial discrimination were positively related to depression (*p* < 0.001), controlling for all sociodemographic factors.
Johnson Nicholson [127]	2020	Cross-sectional	AA; 66%; 60–90; 80	older African American adults	childhood socioeconomic status, financial resources, social resources	For African American older adults, increased social resources were associated with lower depressive symptoms. Adult financial resources were negatively correlated with depressive symptoms (r = −0.19, *p* < 0.05) compared to financial resources during childhood, which were not significantly correlated with depressive symptoms (r = −0.10, *p* = 0.29).
Johnson- Lawrence [128]	2019	Cross-sectional	AA; 35.4%; 25–50; 36	middleclass neighborhoods	educational attainment, discrimination	Higher discrimination was associated with higher depression scores. For men, completing any college was a protective factor, mediating the effects of discrimination and depression. Higher education was inversely associated with depression scores for women, and college did not mediate the effects of discrimination on depression.
Lee [129]	2020	Longitudinal	AA; 51.8%; 19–34; --		perceived racial discrimination	African Americans in the moderate-declining perceived racial discrimination trajectory (growth curve) reported the highest level of depressive symptoms (M = 0.443, SD = 0.579), followed by the high-stable trajectory (M = 0.475, SD = 0.529), low-rising trajectory (M = 0.443, SD = 0.606), and low-stable trajectory (M = 0.345, SD = 0.362). Depressive symptoms differed between members in the moderate-declining and low-stable trajectory.
Lee [130]	2018	Cross-sectional	AA; 54.5%; 21–23; 22.05	emergent adults	racial discrimination	Increased exposure to discrimination led to greater depression symptoms (b = 0.15, 95% C.I. [0.05, 0.24]). There was no affect between discrimination experience and cortisol levels observed through depressive symptoms (b = 0.09, 95% C.I. [−0.18, 0.50]).
Madubata [131]	2018	Cross-sectional	AA; 59.4%; 18–35; 21.4	University students	perceived discrimination	There was a significant positive indirect effect of perceived racial discrimination on depressive symptoms via helplessness (*p* < 0.001). Overall, perceived racial discrimination was significantly associated with depression.
Malcome [132]	2019	Cross-sectional	Black; 100%; 19–60; 38.6	women who have been paroled	social support	Emotional/informational support (*p* < 0.001), tangible support (*p* < 0.001) and positive social interactions (*p* < 0.046) were found to have a significant interaction between recent stressful life events and depressive symptoms; affectionate social support was not statistically significant in relation to recent stressful life events and depressive symptoms.
Mama [133]	2016	Cross-sectional	AA; 74.6%; --; 45.2	African American churchgoers in Houston, TX	subjective social status (community and us), perceived social support	Lower subjective social status and perceived social support were associated with greater depressive symptoms.
Millender [134]	2020	Longitudinal	AA, Black; 100%; 21–46; 31.29	young, socioeconomically disadvantaged mothers	perceived discrimination (racial and major discrimination)	Among young, socioeconomically disadvantaged AA mothers, perceived discrimination (racial and major discrimination subscales) is associated with higher reported symptoms of depression, and major discrimination subscale scores were significantly associated with higher initial depression symptoms. There were no significant changes in depressions symptomology over time except in relation to age, which was associated with a higher depression score.
Mouzon [135]	2017	Cross-sectional	AA; 59.26%; 55–93; 66.65		everyday discrimination	Racial and non-racial everyday discrimination were associated with higher depressive symptoms. Relative to older adults who perceived less overall everyday discrimination, those with higher levels of overall everyday discrimination also had elevated levels of depressive symptoms.
Mugoya [136]	2020	Cross-sectional	AA; 100%; --; 38.9		intimate partner violence, educational attainment	Severe intimate partner violence was significantly associated with increased likelihood of depressive symptoms. Lower educational attainment and receipt of economic assistance were significantly associated with depressive symptoms.
Nguyen [137]	2019	Cross-sectional	AA; 55.97%; 18–93; 43.15		education level, social support	Subjective closeness with friends was negatively associated with MDD. Frequency of contact with friends was negatively associated with MDD among high education respondents but unrelated to MDD among low education respondents. Receipt and provision of support from friends were negatively associated with MDD among high education respondents but positively associated with MDD among low education respondents.
Nowak [138]	2020	Cross-sectional	AA; 100%; 18–45; 27	new mothers	social disorder	Low levels of neighborhood social disorder during pregnancy were associated with higher levels of depressive symptoms for women who reported higher levels of childhood neighborhood social disorder (mean CES-D = 15.47), compared with women who reported lower levels of childhood neighborhood social disorder (mean CES-D = 13.99). Overall, high levels of neighborhood social disorder during pregnancy were associated with higher levels of depressive symptoms regardless of levels of childhood neighborhood social disorder, while low levels of reported neighborhood social disorder in both childhood and pregnancy were associated with the lowest levels of depressive symptoms.
Ong and Burrow [139]	2018	Longitudinal	AA; 76%; 22–52; 30	post-doctoral and doctoral students	daily racial discrimination	More frequent racial discrimination was associated with higher initial depressive symptoms; however, both increased negative affect and decreased positive affect (increased affective reactivity) to daily racial discrimination predicted elevated depressive symptoms independent of discrimination frequency, typical levels of daily negative affect and positive affect, and individual differences in stigma consciousness.
Patterson [140]	2020	Cross-sectional	AA; 100%; 18–93; 42.64	Never incarcerated AA women	familial incarceration	Across social role configurations, familial incarceration was associated with increased depressive symptoms (mean = 9.31; SD = 5.94; *p* < 0.05). Among women with familial incarcerations, those that were “parent only” reported the highest levels of depressive symptoms, while “employed” women reported the lowest levels.
Pickover [141]	2021	Longitudinal	AA, Black; 100%; 18–56; 38.62	low income and IPV exposed	neighborhood disorder, social support	While demonstrating no independent significant effects, the interaction of neighborhood disorder and family social support were associated with higher levels of depression among female survivors of IPV. Higher levels of social support buffered against the negative effects of high neighborhood disorder, but lower levels of social support showed no significant association.
Qin [142]	2020	Longitudinal	AA; 61.1%; 65–100; 73		everyday discrimination, frequency of social contact, perceived social support	Significant association was found between everyday discrimination and depressive symptoms. Each unit of increase in discrimination score predicted 1.24 times the rate of depressive symptoms over time. No significant association was found between frequency of contact and perceived social support and depressive symptoms. Interaction between discrimination and perceived social support from friends as well as family achieved statistical significance in predicting depressive symptoms. For those who reported high levels of social support from friends and family, more frequent experiences of discrimination were associated with more depressive symptoms over time.
Russell [143]	2018	Longitudinal	AA; 100%; 25–80; 37		financial strain, community social disorder, community cohesion, community racism, relationship quality, racism	Neighborhood racial discrimination was significantly related to the development of MDD, and predicted 21% of the variation in depression rates at the neighborhood level over time. Development of MDD was also significantly related to the level of financial problems reported at the time of Wave 1 interviews. Social support was negatively related to subsequent MDD. Additionally, there was also significant association between personal experiences of racism and development of MDD. Interaction between neighborhood racism and relationship quality (social support) was statistically significant, as higher quality relationships reduced the negative effects of neighborhood racism on MDD development.
Sealy- Jefferson [144]	2016	Cross-sectional	AA; 100%; --; 25	postpartum women with 12 years of education or less residing in Detroit, MI	residential environment	Lower perceived neighborhood safety was significantly associated with higher levels of depressive symptoms (which in turn was associated with higher pre-term delivery rates). There was no association found between perceived walkability, food availability, or social disorder and depression.
Tabet [145]	2017	Cross-sectional	AA, Black; 59.12%; 59–74; --	residents of 483 neighborhood blocks in impoverished areas of Saint Louis, MO, or other suburban areas northwest of city	neighborhood conditions	Residing in a neighborhood with adverse conditions for <5 years was associated with non-statistically significantgreater depressive symptoms. In contrast, residing in neighborhoods with adverse conditions for ≥5 years was associated with significantly lower depressive symptoms.
Tamura [146]	2020	Cross-sectional	AA; 6419%; --; 52.6	residents of tri-county area of Jackson, MS	neighborhood violence, neighborhood problems. neighborhood social cohesion	Perceived neighborhood violence and perceived neighborhood problems were associated with higher depressive symptoms scores in both the age- and fully adjusted models. Perceived social cohesion, however, was associated with lower depressive symptoms scores in the age-adjusted model, but showed no significant relationship in the fully adjusted model.
Thomas Tobin and Moody [147]	2021	Cross-sectional	Black; 54.94%; 21–69; 43.52	residents of Nashville and surrounding areas within Davidson County, Tennessee	early life racial discrimination, adult racial discrimination	Individuals who experienced childhood ELRD had 88% lower odds of adult MDD than those who reported none. Adolescent ELRD was linked to nearly 3x greater odds of adult MDD, although this relationship was not significant at *p* < 0.05 (OR = 2.59). Those reporting no ELRD had similar odds of adult MDD to those experiencing racial discrimination later in life. Neither adult major or everyday discrimination was significantly associated with MDD. After controlling for major and everyday adult discrimination, childhood ELRD was associated with significantly lower odds of adult MDD than no ELRD, adolescent ELRD, or adult racial discrimination (OR = 0.05, *p* = 0.02).
Tsuchiya [148]	2018	Cross-sectional	AA; 0%; 22–63; 37.2	non-resident fathers located in two Midwestern industrial cities	perceived financial strain, employment, education, perceived neighborhood conditions, interpersonal relationship stress, social support	Interpersonal stress and perceived financial strain were found to be positively correlated with depressive symptoms, while social support, history of living with son, education level, and employment status were negatively correlated with depressive symptoms. Social support was found to moderate the relationship between interpersonal stress and depressive symptoms.
Watson- Singleton [149]	2019	Cross-sectional	AA, Black; 70%; 18–53; 20.99	students enrolled in a large, predominantly white, urban, university in the southeast	past racial and ethnic discrimination	Past discrimination was positively associated with depressive symptoms.
Weaver [150]	2018	Cross-sectional	AA; 67.6%; 18–92; 45.9	reside in southern rural setting in	family income, material hardship, educational attainment	Educational attainment was significantly associated with depressive symptoms, as fewer years of education correlated with greater depressive symptoms. Material hardship was also significantly associated with depressive symptoms, with incremental increases in material hardship correlating with a 1.1 factor increase in depressive symptoms.
Wheaton [151]	2018	Cross-sectional	AA; 0%; 18+; --	residents of Nashville, TN, and surrounding metropolitan area	discrimination	Overall, no significant differences in depressive symptoms were found between low, moderate, or high levels of major discrimination. Moderate and high everyday discrimination were significantly associated with greater depressive symptoms but not low everyday discrimination; however, depressive symptoms varied significantly by age. Everyday discrimination, but not major discrimination, was associated with greater depressive symptoms among young and middle-aged men. Both major and everyday discrimination were associated with depressive symptoms for older men.
Williams [152]	2021	Longitudinal	Black; 0%; --; --	freshman and sophomore students at public university in Mid-Atlantic	financial status, social support	Overall, stressful life events and perceived financial status were predictors of depressive symptoms; however, perceived financial status was associated with higher levels of depressive symptoms in year 1 when GPA was not added to the model. Social support was not significantly associated with levels of depressive symptoms.
Wu [153]	2019	Cross-sectional	AA; 71.1%; --; 42.07	members of one church in Houston, TX	neighborhood problems, neighborhood vigilance, perceived racial discrimination	Experiences of racial discrimination were associated with greater depression symptoms (b = 0.20, SE = 0.06, *p* < 0.05), but neighborhood problems (b = 0.20, SE = 0.11, *p* = 0.07) and neighborhood vigilance (b = −0.01, SE = 0.04, *p* = 0.74) were not significantly associated with depression symptoms.
Yoon [154]	2019	Cross-sectional	AA; 67.8%; 65–89; 74		perceived discrimination	For older men, depressive symptomology was significantly associated with everyday discrimination. This association was mediated by self-acceptance.

## Appendix B. Full PubMed Search String

(Built Environment [MeSH] OR Employment [MeSH] OR Environmental Exposure [MeSH] OR Food Supply [MeSH] OR Health Literacy [MeSH] OR Health Services Accessibility [MeSH] OR Health Status Disparities [MeSH] OR Homeless Persons [MeSH] OR Hunger [MeSH] OR Insurance [MeSH] OR Internet Access [MeSH] OR Medically Uninsured [MeSH] OR Occupational Stress [MeSH] OR Quality of Health Care [MeSH] OR Residence Characteristics [MeSH] OR Socioeconomic Factors [MeSH] OR Social Behavior [MeSH] OR Social Determinants of Health [MeSH] OR Social Problems [MeSH] OR Social Support [MeSH] OR academic environment [TIAB] OR access to care [TIAB] OR access to health care [TIAB] OR access to therap* [TIAB] OR access to treatment* [TIAB] OR adverse childhood experience* [TIAB] OR built environment [TIAB] OR crime [TIAB] OR discrimination [TIAB] OR economic stabilit* [TIAB] OR economic instabilit* [TIAB] OR economic status [TIAB] OR education access [TIAB] OR education attainment [TIAB] OR educational attainment [TIAB] OR education inequalit* [TIAB] OR educational inequalit* [TIAB] OR education level [TIAB] OR educational status [TIAB] OR employment status [TIAB] OR environmental conditions [TIAB] OR environmental exposure* [TIAB] OR eviction* [TIAB] OR food access [TIAB] OR food deprivation [TIAB] OR food environment [TIAB] OR food insecurit* [TIAB] OR food quality [TIAB] OR food security [TIAB] OR food supply [TIAB] OR health care access [TIAB] OR health care delivery [TIAB] OR health care inequalit* [TIAB] OR health disparit* [TIAB] OR healthcare inequalit* [TIAB] OR healthcare access [TIAB] OR healthcare delivery [TIAB] OR healthcare disparit* [TIAB] OR health services accessibility [TIAB] OR health status disparities* [TIAB] OR homeless* [TIAB] OR housing [TIAB] OR hunger [TIAB] OR illitera* [TIAB] OR incarcerat* [TIAB] OR income [TIAB] OR insurance [TIAB] OR internet access [TIAB] OR language proficien* [TIAB] OR literacy [TIAB] OR living standard* [TIAB] OR medication access [TIAB] OR neighborhood* [TIAB] OR occupational exposure* [TIAB] OR occupational status [TIAB] OR occupational stress [TIAB] OR overcrowding [TIAB] OR poverty [TIAB] OR prejudice [TIAB] OR quality of health care [TIAB] OR race relations [TIAB] OR racial disparities [TIAB] OR racism [TIAB] OR residence characteristics [TIAB] OR segregation [TIAB] OR sense of community [TIAB] OR social behavior* [TIAB] OR social capital [TIAB] OR social class [TIAB] OR social cohesion [TIAB] OR social conditions [TIAB] OR social determinants of health [TIAB] OR social exclusion [TIAB] OR social factor* [TIAB] OR social inequalit* [TIAB] OR social isolation [TIAB] OR social problems [TIAB] OR social support [TIAB] OR socioeconomic factor* [TIAB] OR socioeconomic status [TIAB] OR standard of living [TIAB] OR transportation access [TIAB] OR unemployment [TIAB] OR uninsured [TIAB] OR violence [TIAB] OR working conditions [TIAB] OR workplace conditions [TIAB])

AND

(Behavioral Symptoms [MeSH] OR Mental Disorders [MeSH] OR Mental Health [MeSH] OR Mentally Ill Persons [MeSH] OR Posttraumatic Growth, Psychological [MeSH] OR Psychological Distress [MeSH] OR Psychosocial Functioning [MeSH] OR Resilience, Psychological [MeSH] OR Stress, Psychological [MeSH] OR addiction* [TIAB] OR anxiety [TIAB] OR behavioral health [TIAB] OR behavioral symptom* [TIAB] OR chemical dependen* [TIAB] OR depress* [TIAB] OR emotional wellbeing [TIAB] or emotional well-being [TIAB] OR mental disorder* [TIAB] OR mental health [TIAB] OR mental illness [TIAB] OR mental wellbeing [TIAB] OR mental well-being [TIAB] OR posttraumatic [TIAB] OR post-traumatic [TIAB] OR psychiat* [TIAB] OR psycholog* [TIAB] OR psychosocial [TIAB] OR PTSD [TIAB] OR substance abuse [TIAB] OR substance use [TIAB] OR substance dependen* [TIAB] OR substance related disorder* [TIAB] OR suicid* [TIAB])

AND

(African Americans [MeSH] OR African American* [TIAB] OR Black* [TIAB])

AND

(United States [MeSH] OR united states [TIAB] OR usa [TIAB] OR u.s.a. [TIAB] OR America* [TIAB] OR Appalachia* [TIAB] OR great lakes [TIAB] OR mid-atlantic-state* [TIAB] OR mid-atlantic-region* [TIAB] OR middle-atlantic-state* [TIAB] OR middle-atlantic-region* [TIAB] OR midwestern-us* [TIAB] OR midwestern-u.s* [TIAB] OR Midwestern-state* [TIAB] OR Midwest-state* [TIAB] OR Midwest-us* [TIAB] OR Midwest-u.s* [TIAB] OR great plains [TIAB] OR heartland [TIAB] OR new england [TIAB] OR northeastern-us* [TIAB] OR northeastern-u.s* [TIAB] OR northeastern-state* [TIAB] OR northeast-state* [TIAB] OR northeast-us* [TIAB] OR northeast-u.s* [TIAB] OR pacific northwest [TIAB] OR northwestern-us* [TIAB] OR northwestern-u.s* [TIAB] OR northwest-u.s* [TIAB] OR northwest-us* [TIAB] OR northwestern-state* [TIAB] OR northwest-state* [TIAB] OR pacific-state* [TIAB] OR southeast-state* [TIAB] OR southeastern-state* [TIAB] OR southeast-region [TIAB] OR southeastern-region [TIAB] OR southeast-us* [TIAB] OR southeastern-us* [TIAB] OR southeast-u.s* [TIAB] OR southeastern-u.s* [TIAB] OR southern-state* [TIAB] OR southern-us* [TIAB] OR southern-u.s* [TIAB] OR southwest-state* [TIAB] OR southwestern-state* [TIAB] OR southwest-us* [TIAB] OR southwestern-us* [TIAB] OR southwest-u.s* [TIAB] OR southwestern-u.s* [TIAB] OR deep south [TIAB] OR black belt [TIAB] OR rust belt [TIAB] OR district of Columbia [TIAB] OR Alabama [TIAB] OR Alaska [TIAB] OR Arizona [TIAB] OR Arkansas [TIAB] OR California [TIAB] OR Colorado [TIAB] OR Connecticut [TIAB] OR Delaware [TIAB] OR Florida [TIAB] OR Georgia [TIAB] OR Hawaii [TIAB] OR Hawai’i [TIAB] OR Idaho [TIAB] OR Illinois [TIAB] OR Indiana [TIAB] OR Iowa [TIAB] OR Kansas [TIAB] OR Kentucky [TIAB] OR Louisiana [TIAB] OR Maine [TIAB] OR Maryland [TIAB] OR Massachusetts [TIAB] OR Michigan [TIAB] OR Minnesota [TIAB] OR Mississippi [TIAB] OR Missouri [TIAB] OR Montana [TIAB] OR Nebraska [TIAB] OR Nevada [TIAB] OR New Hampshire [TIAB] OR New Jersey [TIAB] OR New Mexico [TIAB] OR New York [TIAB] OR North Carolina [TIAB] OR North Dakota [TIAB] OR Ohio [TIAB] OR Oklahoma [TIAB] OR Oregon [TIAB] OR Pennsylvania [TIAB] OR Rhode Island [TIAB] OR South Carolina [TIAB] OR South Dakota [TIAB] OR Tennessee [TIAB] OR Texas [TIAB] OR Utah [TIAB] OR Vermont [TIAB] OR Virginia [TIAB] OR Washington [TIAB] OR West Virginia [TIAB] OR Wisconsin [TIAB] OR Wyoming [TIAB] OR Birmingham [TIAB] OR Huntsville [TIAB] OR Montgomery [TIAB] OR Anchorage [TIAB] OR Fairbanks [TIAB] OR Phoenix [TIAB] OR Tucson [TIAB] OR Flagstaff [TIAB] OR Little Rock [TIAB] OR Los Angeles [TIAB] OR San Diego [TIAB] OR San Francisco [TIAB] OR Berkeley [TIAB] OR Stanford [TIAB] OR Denver [TIAB] OR Farmington [TIAB] OR new haven [TIAB] OR Hartford [TIAB] OR Wilmington [TIAB] OR Newark [TIAB] OR Miami [TIAB] OR Gainesville [TIAB] OR Jacksonville [TIAB] OR Tampa [TIAB] OR Tallahassee [TIAB] OR Atlanta [TIAB] OR Honolulu [TIAB] OR Boise [TIAB] OR Chicago [TIAB] OR Urbana [TIAB] OR Evanston [TIAB] OR Indianapolis [TIAB] OR West Lafayette [TIAB] OR Wichita [TIAB] OR Lexington [TIAB] OR Louisville [TIAB] OR New Orleans [TIAB] OR baton rouge [TIAB] OR Shreveport [TIAB] OR Bethesda [TIAB] OR Baltimore [TIAB] OR Boston [TIAB] OR Harvard [TIAB] OR Burlington [TIAB] OR Detroit [TIAB] OR Ann Arbor [TIAB] OR Minneapolis [TIAB] OR Rochester [TIAB] OR St Paul [TIAB] OR Bozeman [TIAB] OR Missoula [TIAB] OR Omaha [TIAB] OR Las Vegas [TIAB] OR Asheville [TIAB] OR Columbus [TIAB] OR Cleveland [TIAB] OR Cincinnati [TIAB] OR Oklahoma [TIAB] OR Portland [TIAB] OR Philadelphia [TIAB] OR Providence [TIAB] OR Charleston [TIAB] OR Nashville [TIAB] OR Memphis [TIAB] OR Houston [TIAB] OR Dallas [TIAB] OR Seattle [TIAB])

NOT ((infant [MeSH] OR child [MeSH] OR adolescent [MeSH]) NOT (adult [MeSH]))

## References

[1] American Psychological Association (2013). Diagnostic and Statistical Manual of Mental Disorders.

[2] James S.L., Abate D., Abate K.H., Abay S.M., Abbafati C., Abbasi N., Abbastabar H., Abd-Allah F., Abdela J., Abdelalim A. (2018). Global, regional, and national incidence, prevalence, and years lived with disability for 354 diseases and injuries for 195 countries and territories, 1990–2017: A systematic analysis for the Global Burden of Disease Study 2017. Lancet.

[3] Bradvik L. (2018). Suicide risk and mental disorders. Int. J. Environ. Res. Public Health.

[4] Reinert M., Nguyen T., Fritze D. (2020). 2021: The State of Mental Health in America.

[5] Cook B.L., Trinh N.H., Li Z., Hou S.S., Progovac A.M. (2017). Trends in racial-ethnic disparities in access to mental health care, 2004-2012. Psychiatr. Serv..

[6] Villarroel M.A., Terlizzi E.P. (2020). Symptoms of Depression Among Adults: United States, 2019.

[7] U.S. Census Bureau. Household Pulse Survey. https://www.cdc.gov/nchs/covid19/pulse/mental-health.htm.

[8] Office of Disease Prevention and Health Promotion Healthy People 2030 Framework. https://health.gov/healthypeople/about/healthy-people-2030-framework.

[9] Office of Disease Prevention and Health Promotion Social Determinants of Health. https://health.gov/healthypeople/objectives-and-data/social-determinants-health.

[10] Williams D.R., Etkins O.S. (2021). Racism and mental health. World Psychiatry.

[11] Pieterse A.L., Todd N.R., Neville H.A., Carter R.T. (2012). Perceived racism and mental health among Black American adults: A meta-analytic review. J. Couns. Psychol..

[12] Williams D.R., Williams-Morris R. (2000). Racism and mental health: The African American experience. Ethn. Health.

[13] Britt-Spells A.M., Slebodnik M., Sands L.P., Rollock D. (2018). Effects of perceived discrimination on depressive symptoms among Black men residing in the United States: A meta-analysis. Am. J. Mens. Health.

[14] Miller J.A. (2021). Examining the relationship between discrimination and depressive symptomatology in African American men: A literature review utilizing a metanarrative approach. J. Am. Psychiatr. Nurses Assoc..

[15] Watkins D.C., Green B.L., Rivers B.M., Rowell K.L. (2006). Depression and Black men: Implications for future research. J. Men’s Health Gend..

[16] Ward E., Mengesha M. (2013). Depression in African American men: A review of what we know and where we need to go from here. Am. J. Orthopsychiatry.

[17] Plowden K.O., Thompson Adams L., Wiley D. (2016). Black and blue: Depression and African American men. Arch. Psychiatr. Nurs..

[18] McLeod M.N., Heller D., Manze M.G., Echeverria S.E. (2020). Police interactions and the mental health of Black Americans: A systematic review. J. Racial. Ethn. Health Disparities.

[19] Thomas P., Duffrin M., Duffrin C., Mazurek K., Clay S.L., Hodges T. (2020). Community violence and African American male health outcomes: An integrative review of literature. Health Soc. Care Community.

[20] Reed D.D., Stoeffler S.W., Joseph R. (2021). Suicide, race, and social work: A systematic review of protective factors among African Americans. J. Evid. Based Soc. Work (2019).

[21] Arksey H., O’Malley L. (2005). Scoping studies: Towards a methodological framework. Int. J. Soc. Res. Methodol..

[22] Levac D., Colquhoun H., O’Brien K.K. (2010). Scoping studies: Advancing the methodology. Implement Sci..

[23] World Health Organization (2016). International Statistical Classification of Diseases and Related Health Problems.

[24] (2013). EndNote.

[25] (2014). Covidence Systematic Review Software.

[26] (2020). Qualtrics Software.

[27] (2018). Microsoft Excel.

[28] Twenge J.M., Cooper A.B., Joiner T.E., Duffy M.E., Binau S.G. (2019). Age, period, and cohort trends in mood disorder indicators and suicide-related outcomes in a nationally representative dataset, 2005-2017. J. Abnorm. Psychol..

[29] Frankham C., Richardson T., Maguire N. (2020). Psychological factors associated with financial hardship and mental health: A systematic review. Clin. Psychol. Rev..

[30] Hasin D.S., Sarvet A.L., Meyers J.L., Saha T.D., Ruan W.J., Stohl M., Grant B.F. (2018). Epidemiology of adult DSM-5 major depressive disorder and its specifiers in the United States. JAMA Psychiatry.

[31] Ganson K.T., Tsai A.C., Weiser S.D., Benabou S.E., Nagata J.M. (2021). Job insecurity and symptoms of anxiety and depression among us young adults during COVID-19. J. Adolesc. Health.

[32] McGee R.E., Thompson N.J. (2015). Peer reviewed: Unemployment and depression among emerging adults in 12 states, Behavioral Risk Factor Surveillance System, 2010. Prev. Chronic Dis..

[33] Minh A., Bültmann U., Reijneveld S.A., van Zon S.K., McLeod C.B. (2021). Childhood socioeconomic status and depressive symptom trajectories in the transition to adulthood in the United States and Canada. J. Adolesc. Health.

[34] Bromberger J.T., Schott L.L., Matthews K.A., Kravitz H.M., Harlow S.D., Montez J.K. (2017). Childhood socioeconomic circumstances and depressive symptom burden across 15 years of follow-up during midlife: Study of Women’s Health Across the Nation (SWAN). Arch. Women’s Ment. Health.

[35] Chen R., Kessler R.C., Sadikova E., NeMoyer A., Sampson N.A., Alvarez K., Vilsaint C.L., Green J.G., McLaughlin K.A., Jackson J.S. (2019). Racial and ethnic differences in individual-level and area-based socioeconomic status and 12-month DSM-IV mental disorders. J. Psychiatr. Res..

[36] Hudson D.L., Bullard K.M., Neighbors H.W., Geronimus A.T., Yang J., Jackson J.S. (2012). Are benefits conferred with greater socioeconomic position undermined by racial discrimination among African American men?. J. Men’s Health.

[37] Thomas C.S. (2015). A new look at the Black middle class: Research trends and challenges. Sociol. Focus.

[38] Phelan J.C., Link B.G., Tehranifar P. (2010). Social conditions as fundamental causes of health inequalities: Theory, evidence, and policy implications. J. Health Soc. Behav..

[39] Williams D.R., Yu Y., Jackson J.S., Anderson N.B. (1997). Racial differences in physical and mental health: Socio-economic status, stress and discrimination. J. Health Psychol..

[40] Ettman C.K., Abdalla S.M., Cohen G.H., Sampson L., Vivier P.M., Galea S. (2020). Prevalence of depression symptoms in us adults before and during the COVID-19 pandemic. JAMA Netw. Open.

[41] Assari S. (2018). Educational attainment better protects African American women than African American men against depressive symptoms and psychological distress. Brain Sci..

[42] Cohen A.K., Nussbaum J., Weintraub M.L.R., Nichols C.R., Yen I.H. (2020). Association of adult depression with educational attainment, aspirations, and expectations. Prev. Chronic Dis..

[43] Nguyen T.T., Tchetgen E.J.T., Kawachi I., Gilman S.E., Walter S., Glymour M.M. (2017). The role of literacy in the association between educational attainment and depressive symptoms. SSM-Popul. Health.

[44] Lorant V., Deliège D., Eaton W., Robert A., Philippot P., Ansseau M. (2003). Socioeconomic inequalities in depression: A meta-analysis. Am. J. Epidemiol..

[45] Lurie L.A., Hagen M.P., McLaughlin K.A., Sheridan M.A., Meltzoff A.N., Rosen M.L. (2021). Mechanisms linking socioeconomic status and academic achievement in early childhood: Cognitive stimulation and language. Cogn. Dev..

[46] Rosen M.L., Sheridan M.A., Sambrook K.A., Meltzoff A.N., McLaughlin K.A. (2018). Socioeconomic disparities in academic achievement: A multi-modal investigation of neural mechanisms in children and adolescents. Neuroimage.

[47] Duke N.N. (2020). Adolescent adversity, school attendance and academic achievement: School connection and the potential for mitigating risk. J. Sch. Health.

[48] Fahle E.M., Reardon S.F., Kalogrides D., Weathers E.S., Jang H. (2020). Racial segregation and school poverty in the United States, 1999–2016. Race Soc. Probl..

[49] Logan J.R., Burdick-Will J. (2017). School segregation and disparities in urban, suburban, and rural areas. ANNALS Am. Acad. Political Soc. Sci..

[50] Stanford Center for Education Policy Analysis Racial and Ethnic Achievement Gaps. https://cepa.stanford.edu/educational-opportunity-monitoring-project/achievement-gaps/race/.

[51] Baranyi G., Sieber S., Cullati S., Pearce J.R., Dibben C.J.L., Courvoisier D.S. (2020). The longitudinal associations of perceived neighborhood disorder and lack of social cohesion with depression among adults aged 50 years or older: An individual-participant-data meta-analysis from 16 high-income countries. Am. J. Epidemiol..

[52] Barnett A., Zhang C.J.P., Johnston J.M., Cerin E. (2018). Relationships between the neighborhood environment and depression in older adults: A systematic review and meta-analysis. Int. Psychogeriatr..

[53] Richardson R., Westley T., Gariépy G., Austin N., Nandi A. (2015). Neighborhood socioeconomic conditions and depression: A systematic review and meta-analysis. Soc. Psychiatry Psychiatr. Epidemiol..

[54] Joshi S., Mooney S.J., Rundle A.G., Quinn J.W., Beard J.R., Cerdá M. (2017). Pathways from neighborhood poverty to depression among older adults. Health Place.

[55] Blair A., Ross N.A., Gariepy G., Schmitz N. (2014). How do neighborhoods affect depression outcomes? A realist review and a call for the examination of causal pathways. Soc. Psychiatry Psychiatr. Epidemiol..

[56] Flournoy E.B. (2021). The rising of systemic racism and redlining in the United States of America. J. Soc. Change.

[57] Bailey Z.D., Krieger N., Agénor M., Graves J., Linos N., Bassett M.T. (2017). Structural racism and health inequities in the USA: Evidence and interventions. Lancet.

[58] Williams D.R., Lawrence J.A., Davis B.A. (2019). Racism and health: Evidence and needed research. Annu. Rev. Public Health.

[59] Slutkin G., Ransford C., Zvetina D. (2018). How the health sector can reduce violence by treating it as a contagion. AMA J. Ethics.

[60] Voith L.A., Topitzes J., Berg K.A. (2020). The transmission of violence and trauma across development and environmental contexts: Intimate partner violence from the perspective of men with histories of perpetration. Child Abuse Negl..

[61] Thulin E.J., Heinze J.E., Kusunoki Y., Hsieh H.F., Zimmerman M.A. (2021). Perceived neighborhood characteristics and experiences of intimate partner violence: A multilevel analysis. J. Interpers. Violence.

[62] Evans M.C., Bazargan M., Cobb S., Assari S. (2020). Mental and physical health correlates of financial difficulties among African-American older adults in low-income areas of Los Angeles. Front. Public Health.

[63] Office of Disease Prevention and Health Promotion Discrimination. https://health.gov/healthypeople/objectives-and-data/social-determinants-health/literature-summaries/discrimination.

[64] Yearby R. (2020). Structural racism and health disparities: Reconfiguring the social determinants of health framework to include the root cause. J. Law Med. Ethics.

[65] Jones C.P., Jones C.Y., Perry G.S., Barclay G., Jones C.A. (2009). Addressing the social determinants of children’s health: A cliff analogy. J. Health Care Poor Underserved.

[66] Alang S., McAlpine D., McCreedy E., Hardeman R. (2017). Police brutality and Black health: Setting the agenda for public health scholars. Am. J. Public Health.

[67] Black Lives Matter. About. https://blacklivesmatter.com/about/.

[68] Bor J., Venkataramani A.S., Williams D.R., Tsai A.C. (2018). Police killings and their spillover effects on the mental health of Black Americans: A population-based, quasi-experimental study. Lancet.

[69] Laurencin C.T., Walker J.M. (2020). Racial profiling is a public health and health disparities issue. J. Racial Ethn. Health Disparities.

[70] Bowleg L., del Río-González A.M., Mbaba M., Boone C.A., Holt S.L. (2020). Negative police encounters and police avoidance as pathways to depressive symptoms among us Black men, 2015-2016. Am. J. Public Health.

[71] Archibald P.C. (2018). Criminal justice contact, stressors, and depressive symptoms among Black adults in the United States. Am. J. Crim. Justice.

[72] Feeney B.C., Collins N.L. (2015). A new look at social support: A theoretical perspective on thriving through relationships. Pers. Soc. Psychol. Rev..

[73] Harandi T.F., Taghinasab M.M., Nayeri T.D. (2017). The correlation of social support with mental health: A meta-analysis. Electron Physician.

[74] Kabo F.W., Antonucci T.C., Jackson J.S. (2019). A social relations and networks perspective of depressive symptoms in older African Americans relative to two other ethno-racial groups. Innov. Aging.

[75] Taylor R.J., Chatters L.M., Woodward A.T., Brown E. (2013). Racial and ethnic differences in extended family, friendship, fictive kin and congregational informal support networks. Fam. Relat..

[76] Oman D., Syme S.L. (2018). Weighing the evidence: What is revealed by 100+ meta-analyses and systematic reviews of religion/spirituality and health?. Why Relig. Spiritual. Matter Public Health.

[77] Davenport A.D., McClintock H.F. (2021). Religiosity and attitudes toward treatment for mental health in the Black church. Race Soc. Probl..

[78] Chen Y., Kim E.S., VanderWeele T.J. (2021). Religious-service attendance and subsequent health and well-being throughout adulthood: Evidence from three prospective cohorts. Int. J. Epidemiol..

[79] Besheer Mohamed K.C., Diamant J., Gecewicz C. Faith among Black Americans. https://www.pewforum.org/2021/02/16/faith-among-black-americans/.

[80] Gaskin-Wasson A.L., Walker K.L., Shin L.J., Kaslow N.J. (2018). Spiritual well-being and psychological adjustment: Mediated by interpersonal needs?. J. Relig. Health.

[81] Herren O.M., Burris S.E., Levy S.-A., Kirk K., Banks K.S., Jones V.L., Beard B., Mwendwa D.T., Callender C.O., Campbell A.L. (2019). Influence of spirituality on depression-induced inflammation and executive functioning in a community sample of African Americans. Ethn. Dis..

[82] Paul Wellstone and Pete Domenici Mental Health Parity and Addiction Equity Act of 2008. Pub. L. No. 110–343, 122 Stat. 3765. 2007–2008.

[83] Patient protection and affordable care act of 2012. Pub. L. No. 111-148, 124 Stat. 119. 2009–2010.

[84] (2017). The Doctor Is Out: Continuing Disparities in Access to Mental and Physical Health Care; National Alliance on Mental Illness. https://www.nami.org/Support-Education/Publications-Reports/Public-Policy-Reports/The-Doctor-is-Out.

[85] Alang S.M. (2019). Mental health care among Blacks in America: Confronting racism and constructing solutions. Health Serv. Res..

[86] Manuel J.I. (2018). Racial/ethnic and gender disparities in health care use and access. Health Serv. Res..

[87] Weissman J., Russell D., Jay M., Malaspina D. (2018). Racial, ethnic, and gender disparities in health care access and use among U.S. adults with serious psychological distress. Psychiatr. Serv..

[88] Agency for Healthcare Research and Quality (2020). 2019 National Healthcare Quality and Disparities Report.

[89] Mongelli F., Georgakopoulos P., Pato M.T. (2020). Challenges and opportunities to meet the mental health needs of underserved and disenfranchised populations in the United States. Focus.

[90] Legha R.K., Miranda J. (2020). An anti-racist approach to achieving mental health equity in clinical care. Psychiatr. Clin. North Am..

[91] Ramchand R., Gordon J.A., Pearson J.L. (2021). Trends in suicide rates by race and ethnicity in the United States. JAMA Netw. Open.

[92] Bray M.J.C., Daneshvari N.O., Radhakrishnan I., Cubbage J., Eagle M., Southall P., Nestadt P.S. (2021). Racial differences in statewide suicide mortality trends in maryland during the coronavirus disease 2019 (COVID-19) pandemic. JAMA Psychiatry.

[93] Flanagin A., Frey T., Christiansen S.L. (2021). Updated guidance on the reporting of race and ethnicity in medical and science journals. JAMA.

[94] Friedman D.B., Arent M.A., Yelton B., Sakhuja M., Haynes V.E., Noblet S., Brandt H.M., Isenhower W.D., Wandersman A., Zona D. (2020). Development of a clinical-academic-community collaboration to improve health literacy. J. Prim. Care Community Health.

[95] Jackson J.S., Torres M., Caldwell C.H., Neighbors H.W., Nesse R.M., Taylor R.J., Trierweiler S.J., Williams D.R. (2004). The National Survey of American Life: A study of racial, ethnic and cultural influences on mental disorders and mental health. Int. J. Methods Psychiatr. Res..

[96] Redwood Y., Schulz A.J., Israel B.A., Yoshihama M., Wang C.C., Kreuter M. (2010). Social, economic, and political processes that create built environment inequities: Perspectives from urban African Americans in Atlanta. Fam. Community Health.

[97] Orgera K., Garfield R., Rudowitz R. Tracking Social Determinants of Health during the COVID-19 Pandemic. https://www.kff.org/coronavirus-covid-19/issue-brief/tracking-social-determinants-of-health-during-the-covid-19-pandemic/.

[98] McKnight-Eily L.R., Okoro C.A., Strine T.W., Verlenden J., Hollis N.D., Njai R., Mitchell E.W., Board A., Puddy R., Thomas C. (2021). Racial and ethnic disparities in the prevalence of stress and worry, mental health conditions, and increased substance use among adults during the COVID-19 pandemic—United States, April and May 2020. MMWR Morb. Mortal Wkly Rep..

[99] Alexander K.A., Willie T.C., McDonald-Mosley R., Campbell J.C., Miller E., Decker M.R. (2021). Associations between reproductive coercion, partner violence, and mental health symptoms among young Black women in Baltimore, Maryland. J. Interpers. Violence.

[100] Amutah-Onukagha N.N., Doamekpor L.A., Gardner M. (2017). An examination of the sociodemographic and health determinants of major depressive disorder among Black women. J. Racial Ethn. Health Disparities.

[101] Assari S., Cobb S., Saqib M., Bazargan M. (2020). Economic strain deteriorates while education fails to protect Black older adults against depressive symptoms, pain, self-rated health, chronic disease, and sick days. J. Ment. Health Clin. Psychol..

[102] Assari S., Lankarani M.M., Caldwell C.H. (2018). Does discrimination explain high risk of depression among high-income African American men?. Behav. Sci..

[103] Assari S., Miller R.J., Taylor R.J., Mouzon D., Keith V., Chatters L.M. (2018). Discrimination fully mediates the effects of incarceration history on depressive symptoms and psychological distress among African American men. J. Racial Ethn. Health Disparities.

[104] Baldwin-Clark T., Ofahengaue Vakalahi H.F., Anderson B. (2016). What about African American older women and depressive symptoms?. Educ. Gerontol..

[105] Benca-Bachman C.E., Najera D.D., Whitfield K.E., Taylor J.L., Thorpe R.J., Palmer R.H.C. (2020). Quality and quantity of social support show differential associations with stress and depression in African Americans. Am. J. Geriatr. Psychiatry.

[106] Bottomley J.S., Burke L.A., Neimeyer R.A. (2017). Domains of social support that predict bereavement distress following homicide loss: Assessing need and satisfaction. Omega J. Death Dying.

[107] Brooks J.R., Hong J.H., Madubata I.J., Odafe M.O., Cheref S., Walker R.L. (2020). The moderating effect of dispositional forgiveness on perceived racial discrimination and depression for African American adults. Cult. Divers. Ethn. Minority Psychol..

[108] Bukowski L.A., Hampton M.C., Escobar-Viera C.G., Sang J.M., Chandler C.J., Henderson E., Creasy S.L., Stall R.D. (2019). Intimate partner violence and depression among Black transgender women in the USA: The potential suppressive effect of perceived social support. J. Urban Health.

[109] Caldwell C.H., Misra D.P., Rogers W.B., Young A., Giurgescu C. (2018). Interpersonal relationships among Black couples and depressive symptoms during pregnancy. MCN: Am. J. Matern. Child Nurs..

[110] Chae D.H., Powell W.A., Nuru-Jeter A.M., Smith-Bynum M.A., Seaton E.K., Forman T.A., Turpin R., Sellers R. (2017). The role of racial identity and implicit racial bias in self-reported racial discrimination: Implications for depression among African American men. J. Black Psychol..

[111] Chang E.C., Chang O.D., Rollock D., Lui P.P., Watkins A.F., Hirsch J.K., Jeglic E.L. (2019). Hope above racial discrimination and social support in accounting for positive and negative psychological adjustment in African American adults: Is ’knowing you can do it’ as important as ’knowing how you can’?. Cogn. Ther. Res..

[112] Chatters L.M., Nguyen A.W., Taylor R.J., Hope M.O. (2018). Church and family support networks and depressive symptoms among African Americans: Findings from the National Survey of American Life. J. Community Psychol..

[113] Christie-Mizell C.A., Talbert R.D., Hope A.R., Frazier C.G., Hearne B.N. (2019). Depression and African Americans in the first decade of midlife: The consequences of social roles and gender. J. Natl. Med. Assoc..

[114] Clark E.M., Williams R.M., Schulz E., Williams B.R., Holt C.L. (2018). Personality, social capital, and depressive symptomatology among African Americans. J. Black Psychol..

[115] English D., Hickson D.A., Callander D., Goodman M.S., Duncan D.T. (2020). Racial discrimination, sexual partner race/ethnicity, and depressive symptoms among Black sexual minority men. Arch. Sex Behav..

[116] Evans M.C., Cobb S., Smith J., Bazargan M., Assari S. (2019). Depressive symptoms among economically disadvantaged African American older adults in south los angeles. Brain Sci..

[117] Gayman M.D., Kail B.L., Spring A., Greenidge G.R. (2018). Risk and protective factors for depressive symptoms among African American men: An application of the stress process model. J. Gerontol. Ser. B Psychol. Sci. Soc. Sci..

[118] Goodwill J.R., Taylor R.J., Watkins D.C. (2021). Everyday discrimination, depressive symptoms, and suicide ideation among African American men. Arch. Suicide Res..

[119] Hart A.R., Lavner J.A., Carter S.E., Beach S.R.H. (2021). Racial discrimination, depressive symptoms, and sleep problems among Blacks in the rural south. Cult. Divers. Ethn. Minority Psychol..

[120] Hawkins M., Misra D., Zhang L., Price M., Dailey R., Giurgescu C. (2020). Family involvement in pregnancy and psychological health among pregnant Black women. Arch. Psychiatr. Nurs..

[121] Heldreth C.M., Guardino C.M., Wong L.H., Schetter C.D., Shapiro J.R., Schafer P., Shalowitz M., Lanzi R.G., Thorp J., Raju T. (2016). Childhood racism experiences and postpartum depressive symptoms in African American mothers. J. Soc. Clin. Psychol..

[122] Hoggard L.S., Powell W., Upton R., Seaton E., Neblett E.W. (2019). Racial discrimination, personal growth initiative, and African American men’s depressive symptomatology: A moderated mediation model. Cult. Divers. Ethn. Minority Psychol..

[123] Hoggard L.S., Volpe V., Thomas A., Wallace E., Ellis K. (2019). The role of emotional eating in the links between racial discrimination and physical and mental health. J. Behav. Med..

[124] Holmes S.C., Ciarleglio M.M., Song X., Clayton A., Smith M.V. (2020). Testing the family stress model among Black women receiving Temporary Assistance for Needy Families (TANF). J. Child Fam. Stud..

[125] Holt C.L., Roth D.L., Huang J., Clark E.M. (2018). Role of religious social support in longitudinal relationships between religiosity and health-related outcomes in African Americans. J. Behav. Med..

[126] Hudson D.L., Neighbors H.W., Geronimus A.T., Jackson J.S. (2016). Racial discrimination, John Henryism, and depression among African Americans. J. Black Psychol..

[127] Johnson Nicholson M.C., Martin P., Gilligan M., Cutrona C.E., Russell D.W., Schofield T.J., Poon L.W. (2020). The impact of distal influences and proximal resources on the mental health of African American older adults: Findings from the Georgia Centenarian Study. Innov. Aging.

[128] Johnson-Lawrence V., Scott J.B., James S.A. (2019). Education, perceived discrimination and risk for depression in a southern Black cohort. Aging Ment. Health.

[129] Lee D.B., Anderson R.E., Hope M.O., Zimmerman M.A. (2020). Racial discrimination trajectories predicting psychological well-being: From emerging adulthood to adulthood. Dev. Psychol..

[130] Lee D.B., Peckins M.K., Heinze J.E., Miller A.L., Assari S., Zimmerman M.A. (2018). Psychological pathways from racial discrimination to cortisol in African American males and females. J. Behav. Med..

[131] Madubata I.J., Odafe M.O., Talavera D.C., Hong J.H., Walker R.L. (2018). Helplessness mediates racial discrimination and depression for African American young adults. J. Black Psychol..

[132] Malcome M.L.D., Fedock G., Garthe R.C., Golder S., Higgins G., Logan T.K. (2019). Weathering probation and parole: The protective role of social support on Black women’s recent stressful events and depressive symptoms. J. Black Psychol..

[133] Mama S.K., Li Y., Basen-Engquist K., Lee R.E., Thompson D., Wetter D.W., Nguyen N.T., Reitzel L.R., McNeill L.H. (2016). Psychosocial mechanisms linking the social environment to mental health in African Americans. PLoS ONE.

[134] Millender E., Barile J.P., Bagneris J.R., Harris R.M., De Faria L., Wong F.Y., Crusto C.A., Taylor J.Y. (2020). Associations between social determinants of health, perceived discrimination, and body mass index on symptoms of depression among young African American mothers. Arch. Psychiatr. Nurs..

[135] Mouzon D.M., Taylor R.J., Keith V.M., Nicklett E.J., Chatters L.M. (2017). Discrimination and psychiatric disorders among older African Americans. Int. J. Geriatr. Psychiatry.

[136] Mugoya G.C.T., Witte T., Bolland A., Tomek S., Hooper L.M., Bolland J., George Dalmida S. (2020). Depression and intimate partner violence among African American women living in impoverished inner-city neighborhoods. J. Interpers. Violence.

[137] Nguyen A.W., Walton Q.L., Thomas C., Mouzon D.M., Taylor H.O. (2019). Social support from friends and depression among African Americans: The moderating influence of education. J. Affect Disord..

[138] Nowak A.L., Giurgescu C., Templin T.N., Dailey R.K., Misra D.P. (2020). How depressive symptoms among African American women relate to measures of social disorder in her childhood and pregnancy neighborhood. J. Urban Health.

[139] Ong A.D., Burrow A.L. (2018). Affective reactivity to daily racial discrimination as a prospective predictor of depressive symptoms in African American graduate and postgraduate students. Dev. Psychopathol..

[140] Patterson E.J., Talbert R.D., Brown T. (2020). Familial incarceration, social role combinations, and mental health among African American women. J. Marriage Fam..

[141] Pickover A.M., Bhimji J., Sun S., Evans A., Allbaugh L.J., Dunn S.E., Kaslow N.J. (2021). Neighborhood disorder, social support, and outcomes among violence-exposed African American women. J. Interpers. Violence.

[142] Qin W., Nguyen A.W., Mouzon D.M., Hamler T.C., Wang F. (2020). Social support, everyday discrimination, and depressive symptoms among older African Americans: A longitudinal study. Innov. Aging.

[143] Russell D.W., Clavél F.D., Cutrona C.E., Abraham W.T., Burzette R.G. (2018). Neighborhood racial discrimination and the development of major depression. J. Abnorm. Psychol..

[144] Sealy-Jefferson S., Giurgescu C., Slaughter-Acey J., Caldwell C., Misra D. (2016). Neighborhood context and preterm delivery among African American women: The mediating role of psychosocial factors. J. Urban Health.

[145] Tabet M., Sanders E.A., Schootman M., Chang J.J., Wolinsky F.D., Malmstrom T.K., Miller D.K. (2017). Neighborhood conditions and psychosocial outcomes among middle-aged African Americans. J. Prim. Care Community Health.

[146] Tamura K., Langerman S.D., Orstad S.L., Neally S.J., Andrews M.R., Ceasar J.N., Sims M., Lee J.E., Powell-Wiley T.M. (2020). Physical activity-mediated associations between perceived neighborhood social environment and depressive symptoms among Jackson Heart Study Participants. Int. J. Behav. Nutr. Phys. Act..

[147] Thomas Tobin C.S., Moody M.D. (2021). Does early life racial discrimination explain a mental health paradox among Black adults?. J. Aging Health.

[148] Tsuchiya K., Qian Y., Thomas A., Hill De Loney E., Caldwell C.H. (2018). The effects of multiple dimensions of risk and protective factors on depressive symptoms among nonresident African American fathers. Am. J. Community Psychol..

[149] Watson-Singleton N.N., Hill L.K., Case A.D. (2019). Past discrimination, race-related vigilance, and depressive symptoms: The moderating role of mindfulness. Mindfulness.

[150] Weaver A., Taylor R.J., Chatters L.M., Himle J.A. (2018). Depressive symptoms and psychological distress among rural African Americans: The role of material hardship and self-rated health. J. Affect Disord..

[151] Wheaton F.V., Thomas C.S., Roman C., Abdou C.M. (2018). Discrimination and depressive symptoms among African American men across the adult lifecourse. J. Gerontol. B Psychol. Sci. Soc. Sci..

[152] Williams K.D.A., Adkins A.E., Kuo S.I., LaRose J.G., Utsey S.O., Guidry J.P.D., Dick D., Carlyle K.E. (2021). Risk, protective, and associated factors of anxiety and depressive symptoms and campus health services utilization among Black men on a college campus. J. Racial Ethn. Health Disparities.

[153] Wu I.H.C., Strong L.L., Nguyen N.T., Cho D., John J., McNeill L.H. (2019). Psychosocial stressors, depression, and physical activity among African Americans. Am. J. Health Behav..

[154] Yoon E., Coburn C., Spence S.A. (2019). Perceived discrimination and mental health among older African Americans: The role of psychological well-being. Aging Ment. Health.

